# Combined Experimental and CFD Approach of Two-Phase Flow Driven by Low Thermal Gradients in Wine Tanks: Application to Light Lees Resuspension

**DOI:** 10.3390/foods9070865

**Published:** 2020-07-02

**Authors:** Fabien Bogard, Fabien Beaumont, Yann Vasserot, Florica Simescu-Lazar, Blaise Nsom, Gérard Liger-Belair, Guillaume Polidori

**Affiliations:** 1 Université de Reims Champagne-Ardenne, UFR Sciences Exactes et Naturelles, BP 1039, CEDEX 2, 51687 Reims, France; fabien.bogard@univ-reims.fr (F.B.); fabien.beaumont@univ-reims.fr (F.B.); guillaume.polidori@univ-reims.fr (G.P.); 2 LOCA-LVBE, Université de Reims Champagne-Ardenne, UFR Sciences Exactes et Naturelles, BP 1039, CEDEX 2, 51687 Reims, France; yann.vasserot@univ-reims.fr; 3 Engineering and Materials Science Laboratory, LISM EA 4695, Université de Reims Champagne-Ardenne, BP 1039, CEDEX 2, 51687 Reims, France; florica.lazar@univ-reims.fr; 4 IRDL/UBO UMR CNRS 6027, Université de Bretagne Occidentale, Rue de Kergoat, 29238 Brest, France; blaise.nsom@univ-brest.fr; 5 Equipe Effervescence, Champagne et Applications (GSMA/UMR CNRS 7331), Université de Reims Champagne-Ardenne, UFR Sciences Exactes et Naturelles, BP 1039, CEDEX 2, 51687 Reims, France

**Keywords:** wine, light lees, thermal gradient, two-phase flow, CFD

## Abstract

In winemaking, clarification and stabilization are the processes by which insoluble matter suspended in the wine (called lees) is removed before bottling. The light lees represent 2–4% of the total wine volume. Under certain circumstances, resuspension of lees may occur. The resuspension of lees has been attributed to temperature variations between the wine stored in tanks and the environment of the cellar. From in situ, laboratory-scale studies involving laser tomography techniques, it was shown that low (positive or negative) thermal gradients between a wine tank containing light lees and its external environment induce mass transfer by natural convection. To extrapolate these findings to full-scale tanks, an Eulerian-Eulerian multiphase CFD model was applied to simulate the two-phase flow behavior as a function of temperature variations on a 24–h cycle. Numerical temperature and time-dependent flow patterns of both wine and lees confirm that low thermal gradients induce sufficient fluid energy to resuspend the lees, thus showing that the laboratory results can be extrapolated to full-scale tanks.

## 1. Introduction

In winemaking, so-called lees are generated after the fermentation of grape must. They consist of a solid phase mainly composed of microorganisms (yeast and bacteria), insoluble carbohydrates, phenolic compounds, lignin, proteins, tartaric acid, a liquid phase rich in ethanol and organic acids, colloids and inorganic matter [1,2,3]. Lees are undoubtedly the least exploited byproducts of the winemaking industry [1,4]. Of all the compounds that can be found in lees, polyphenols exhibit powerful bioactive properties. Their extraction may thus be of particular interest, and was explored recently, from red wine lees, via an ultrasound-assisted extraction procedure [4].

In winemaking, the alcoholic fermentation process (usually carried out in tanks) is followed by a period of storage in contact with lees (known as storage on light lees). The lees used for aging wines correspond to only a small proportion of the total quantity produced during wine production. The light lees represent 2–4% of the total wine volume [5]. During aging, the wine is periodically stirred in order to resuspend the lees. It should me mentioned that one of the factors of interest in lees breeding is the release of compounds from lees, such as mannoproteins, compounds that can influence the physical–chemical composition, as well as the organoleptic profile of the final wines. This method, which consists of stirring the light lees of the wine that settle at the bottom of the tanks or barrels during winemaking, is known as *bâtonnage* in French. This process of mechanical mixing is purely empirical and does not comply with any specifications. *Bâtonnage* favors the redistribution of the polysaccharides, amino acids, nucleic acids and esters contained in lees, well known for their strong flavors [3,6]. Although stirring is mainly used for white wines aged on lees, some winegrowers now choose to integrate stirring into their red winemaking itinerary.

Under certain circumstances, the resuspension of lees may also be carried out involuntarily, thus perturbing the winemaking process; this may be the case when the cause for resuspending the lees is not of mechanical (by the stirring action of wine) but of thermal origin. Such circumstances may occur as the wines are left in stainless steel tanks or wooden barrels in nonthermoregulated cellars, which is quite common in the wine world. For example, in November in the Champagne-Ardenne region in France (where the most renowned French sparkling wine is produced), cellars can usually be subjected to wide differences in temperatures, ranging from 4 °C in the morning to 20 °C in the afternoon. And in the near future, global warming will certainly bring about even bigger differences between the day- and night-time temperatures [7]. Under such circumstances, the wine temperature can undergo fluctuations by pure thermal conduction through the container wall. The corresponding thermal fluctuations of a wine stored in a tank or barrel can thus be sufficient to promote free convection inside the container [8]. Nowadays, it should nevertheless be noted that most large wineries use isothermal tanks for wine storage periods in order to avoid such fluctuations. Consequently, such an issue is typically limited to small- and medium-sized wineries.

Under a low thermal gradient, free convection in tanks or wooden vats is the result of differences in wine density in the close vicinity of the reservoir walls, due to the thermal gradients between the wine and the cellar environment [9,10]. Under such circumstances, free convection in wine may bring the fine lees into suspension within the wine bulk. Taken as a whole, the problem of wine storage is complex, and the heat transfer mode is threefold, involving forced convection between the outside and the tank (because wine tanks are generally stored in hangars where draughts are present), conduction in the thickness of the tank walls, and free convection between the inside of the tank and the liquid medium.

The question that motivated this study is whether the free convection driven by the thermal gradient is sufficient to promote an effective stirring effect to resuspend the light lees within the wine bulk. Two distinct methods were applied to answer these questions. As a first step, a purely experimental study was established by creating a low thermal gradient of 3 °C (either positive T_room_ > T_wine_ or negative T_room_ < T_wine_) between the container and the outside of the container. If these low gradients induce a resuspension of the lees, then, by extension, higher gradients will contribute even more. In a second step, a numerical study was carried out to ascertain whether the laboratory results could be extrapolated to full-scale tanks. For this purpose, Computational Fluid Dynamics (CFD) calculations were performed using a calculation code based on the finite volume method and a two-phase flow model (the wine and the light lees, respectively).

## 2. Experimental Procedure: Highlighting the Thermal Resuspension Process of Light Lees

Two simple experiments were carried out in order to determine whether low thermal gradients could be the driving force behind the resuspension of light lees in wine tanks. The first set of experiments focused on characterizing the flow structure solely within the liquid medium, without lees, while the second focused exclusively on lees resuspension. The wine samples with fine lees were obtained after the alcoholic fermentation of a grape juice (originating from the Chardonnay grape variety, grown in the Champagne region, and typically involved in the composition of Champagne wine). The wine was previously clarified by racking to remove heavy lees. Glass cylindrical laboratory receptacles with a capacity of 1 L and a diameter of 10 cm were used for this set of experiments.

### 2.1. Flow Dynamics Within the Wine Bulk

To investigate the natural convection flow patterns, a simple first experiment was conducted using a clear white wine sample at 12 °C, without the presence of lees. The wine sample was subjected to a low positive thermal gradient (with the external environment temperature being slightly higher than that of the wine), and to a negative one (with the external environment temperature being slightly lower than that of wine). Due to the axisymmetric nature of the convective flow, a 2D investigation was sufficient to correctly reveal the dynamics of the overall 3D flow patterns. Natural convection flow can thus be highlighted through laser tomography (with a 2 W argon laser light sheet - INNOVA 70Ce2W), with the laser sheet crossing the plane of symmetry of the cylindrical receptacle [11,12,13,14]. For this purpose, the fluid was seeded with neutrally buoyant glass-hallow spheres (with diameters 9–13 μm, and density close to 1.1 g·cm^−3^) which were likely to produce sufficient light scattering and subsequent facilitate efficient traceability of the flow.

**Figure 1:**
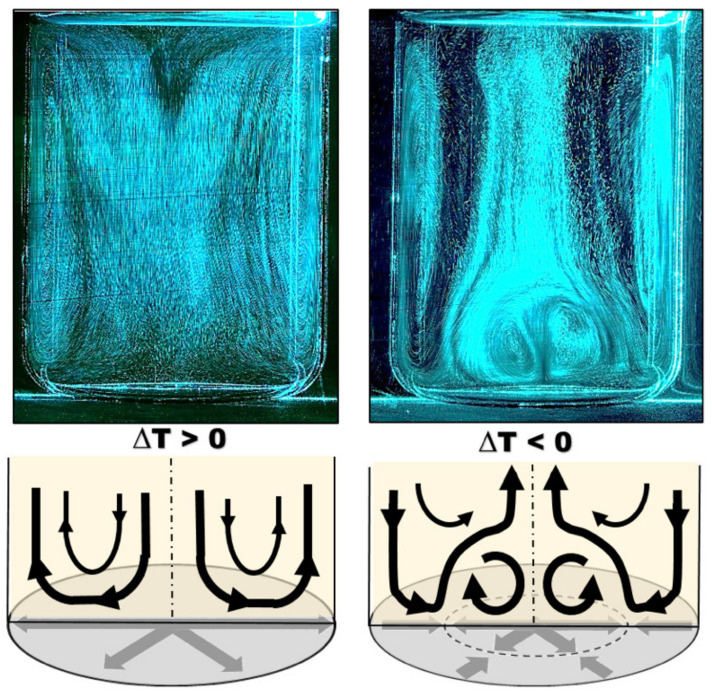
Flow behavior within the wine bulk, and flow directions near the bottom surface of the container, under a low positive thermal gradient of +3 °C (left), and a low negative thermal gradient of −3 °C (right), respectively 15 min after the start of the heating/cooling process of the room.

The flow analysis within the wine bulk resulting from the low thermal gradients between the wine and the external environment is displayed in Figure 1. As shown in Figure 1, the flow dynamics showed radically different overall behavior depending on the thermal gradient direction [15,16,17]. A positive thermal gradient drove overall upward vertical movement of the wine along the receptacle wall. The axisymmetry of the vessel then led to the production of large-scale counter-rotating vortex cells, with a wine column falling along the axis of symmetry of the cylindrical receptacle. The size of these convective cells allowed the entire volume of fluid to be set in motion by viscous drive. It was clear that the lowest flow velocities were in the lower part of the container (where the fine lees, if present, progressively settled). In contrast, a low negative thermal gradient drove a flow with a more complex topology. Natural convection was initially directed downwards along the receptacle wall with two large vortex zones. The singularity of this case was linked to the presence of a vortex zone attached to the bottom of the tank and composed of two counter-rotating vortex cells of higher intensities. We can thus speculate that, depending on the intensity of this bottom vortex, the lees that have settled at the bottom of the receptacle could be more or less easily resuspended within the wine bulk. Moreover, in this thermal configuration, an internal portion of the wine bulk (the dark areas found in Figure 1 on the right) did not appear to be involved in the mixing process.

While Figure 1 clearly shows that natural convection is the driving force behind the wine motion, particular attention must be paid to what is happening at the bottom of the vessel and at the interface between the flow and this bottom. Indeed, it is this interaction that will be the site of the initial movement of the light lees and the process of their resuspension within the container. A summary of the flows specifically found at the bottom of the container, under both thermal configurations, is displayed in Figure 1 (below the respective fluid-tomography images). It is clear that two different topologies are present. On one hand, a positive thermal gradient at the wall resulted in a parietal flow directed from the center of the vessel to the side wall. On the other hand, a negative thermal gradient clearly generated two oppositely-directed flows that met at an intermediate distance between the center of the container and the side wall. These two distinct configurations with regard to the flow dynamics found at the bottom of the container suggest different resuspension behaviors for the light lees that settle near the bottom of the container.

### 2.2. Settling Dynamics of the Light Lees

**Figure 2:**
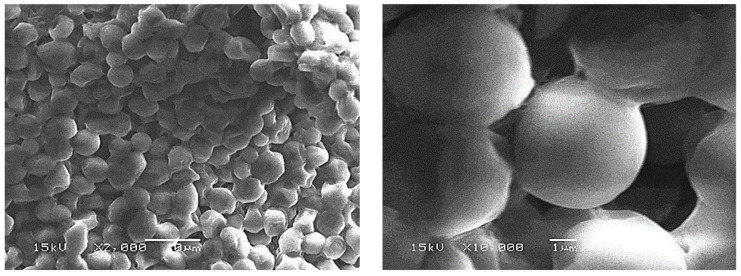
Micrographs taken through Scanning Electron Microscopy (SEM) showing the nearly monodisperse size distribution of light lees (magnification ×2000 left–×10,000 right).

Does the gravitational settling rate of the light lees have an influence on their resuspension in the wine bulk, or is it negligible compared with the characteristic velocity field of the flow patterns driven by the low thermal gradients? To answer this question, a small amount of light lees was collected from the wine. Two micrographs of light lees, taken using a 15kV JEOL/JSM 6460LA scanning electron microscope, are displayed in Figure 2. The lees appeared to be quasi-spherical in shape, with a nearly monodisperse size distribution (close to 5 μm in diameter).

The average density of wine lees is close to 1.25 g·cm^−3^ [6] (i.e., higher than that of the wine). Given that density of lees is significantly higher than that of the liquid phase, it should be considered a serious obstacle to their resuspension in the wine bulk. For such small solid particles, the settling velocity typically obeys the so-called Stokes velocity, according to the following relationship:

(1) $$V_{s}=\frac{gd_{l}^{2}}{18\mu_{w}}\left(\rho_{l}-\rho_{w}\right)$$

with *g*, $d_{l}$, $\rho_{l}$, $\rho_{w}$ and $\mu_{w}$, being respectively the gravity acceleration, the average lees diameter, the density of lees, the density of the wine, and the wine dynamic viscosity.

The temperature dependence of the champagne wine density was determined by Hlavac et al. [15], and is expressed as follows:

(2) $$\rho_{w}=-0.0055\;T^{2}\;\left({}^{\circ}C\right)+0.025\;T\left({}^{\circ}C\right)+999.89$$

The temperature dependence of the wine dynamic viscosity typically follows an Arrhenius-like equation. The following equation was derived by Liger-Belair et al. [18] for a standard Champagne wine free from dissolved CO_2_ (with the temperature *T* being expressed in K):

(3) $$\mu_{w}=1.08\times 10^{-7}\;\exp\left(\frac{2806}{T\;}\right)$$

For light lees with 5 µm in diameter and at a wine temperature of 12 °C, the settling velocity of lees was found to be of 0.0016 mm·s^−1^. By using a particle image velocimetry (PIV) measurement system, including a Litron Nd:YAG laser and a CCD video camera identical to that used in a previous study [19], the velocity field was properly determined in the wine receptacle. The maximum recorded wine velocities were close to 1.2 mm·s^−1^ (i.e., in the order of 10^3^ times higher than the settling velocities) [20]. It can thus be concluded that the gravitational sedimentation rates of the light lees alone cannot influence their resuspension in the wine bulk under the effect of the low thermal gradients.

### 2.3. Resuspension of Light Lees

**Figure 3:**
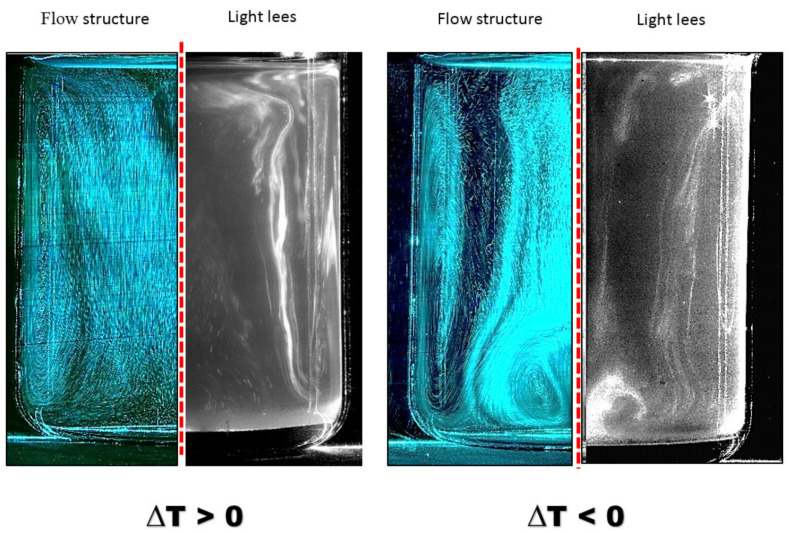
Resuspension process of light lees under low positive thermal gradient +3 °C (left) and low negative thermal gradient −3 °C (right), 15 min after the start of the heating/cooling process of the room.

In order to visualize the resuspension of the lees in the receptacle (filled with 1 L of wine with lees) subjected to the low thermal gradients, the same laser tomography process was used. In a lees-free sample, tracers were added to the liquid phase to serve as wine markers in order to build the tomography-images of the wine flow structures (as described in Section 2.1). In the present situation, the light lees will serve as tracers. Actually, in addition to being neutrally buoyant, light lees produce sufficient light scattering and become clearly visible under laser lighting, as shown in Figure 3. The tomography-images displayed in Figure 3 compare the wine flow structure presented in Section 2.1 with the subsequent resuspension of light lees resulting from the low thermal gradients between the wine and the external environment. It is clearly shown in Figure 3 that whatever the sign of ΔT (i.e., the direction of the thermal gradient), the resuspension of light lees faithfully follows the global structure of the wine flow resulting from the thermal gradient. If the thermal gradient is positive, the lees from the bottom of the vessel are directed upward near the wall to form a main vortex. If the thermal gradient is negative, the lees peel off the bottom of the container in the form of a vortex tongue, before being reinjected into the vessel to form a main vortex opposite to the one described above. Undoubtedly, as this 1 L receptacle was subjected to low thermal gradients, a free convection process was driven, with the ability to resuspend a significant part of the lees which had settled on the bottom. Nevertheless, unlike with efficient mechanical stirring such as *bâtonnage*, a low thermal gradient, i.e., ±3 °C, does not allow all the settled lees to be fully stirred, and therefore efficiently resuspended in the wine bulk. However, we can legitimately speculate that the greater the thermal amplitude, the more efficient the resuspension of the light lees.

## 3. Numerical Procedure: Application of CFD to a Full-Scale Wine Tank

### 3.1. Heat Transfer and Numerical Methods

**Figure 4:**
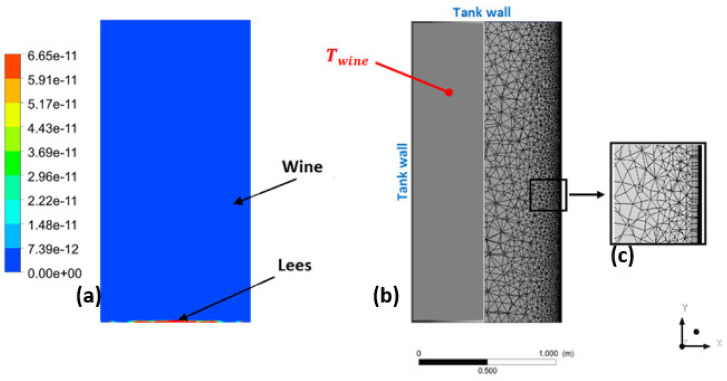
Volume fraction of light lees in the liquid bulk (**a**). Superposition of the geometry of the tank (left) and the mesh of the fluid domain (right) (**b**). Meshing of the fluid domain and detail of the refinement of the mesh close to the wall (**c**).

Computational Fluid Dynamics requires several steps to ensure the accuracy and reliability of the fluid flow analysis. The first step consists of defining the geometry of a full-scale wine tank. A standard cylindrical geometry with a diameter of 1.1 m and a height of 2.2 m (equivalent to that of a standard commercial tank) was used in the following tests. The calculation domain was meshed using the ANSYS Workbench Meshing^®^ software. The mesh consisted of about 15,000 hexahedral and tetrahedral cells. The unstructured mesh was refined on the wall where a high resolution boundary layer is required. while it is coarser in the far field, as seen in Figure 4. The problem is considered to be axisymmetric and time-dependent, but not isothermal.

**Figure 5:**
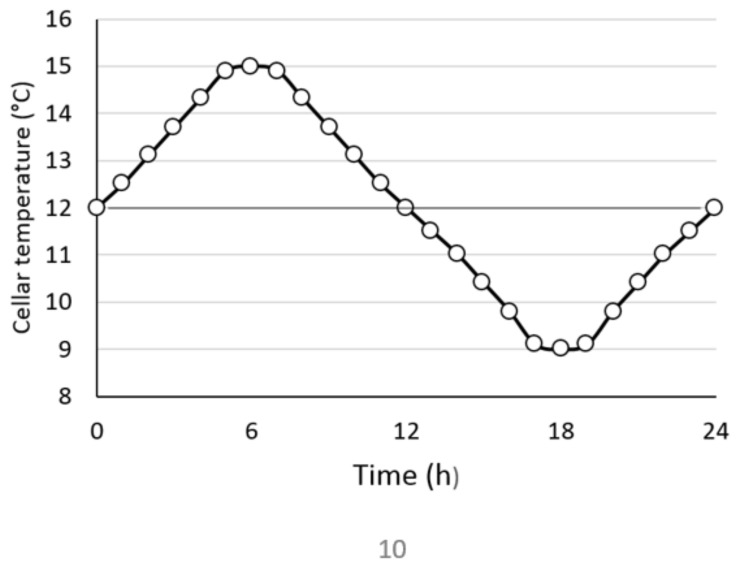
Evolution of the cellar temperature according to time on a 24 h cycle.

The ANSYS Fluent 19.3^®^ commercial code was used to calculate the anisothermal flow in the 2D calculation domain. Convective exchanges require the activation of the energy equation to take into account thermal transfer phenomena. Depending on the wine-growing region, not all wine cellars are thermo-regulated; the temperature of the ambient air in cellars can fluctuate daily [21]. Such fluctuations can thus affect the wine temperature by conduction through the wall of the tanks, vats or barrels. In order to mimic these thermal fluctuations, a subroutine was implemented in the CFD code which modulates the temperature of the tank wall according to the time of the day, according to the scheme displayed in Figure 5.

This subroutine is implemented in the calculation code and modulates the thermal boundary condition at the wall at each time-step of the simulation. In order to enable comparison between the laboratory-scale studies and the numerical simulation carried out on real size tanks, we used an identical temperature gradient of +/−3 °C (i.e., a wine at an initial temperature of 12 °C and a cellar temperature varying from 9–15 °C according to the 24 h cycle displayed in Figure 5). An analytical method, which is fully described below, was used to calculate the thermal boundary condition of the tank wall. The temperature of the inner wall of the tank was calculated from various parameters such as the thickness and material of the tank walls or the external temperature.

**Figure 6:**
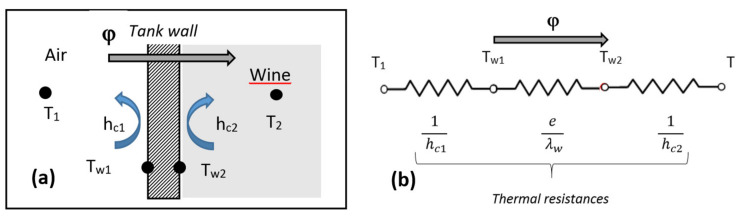
Sketch of the heat transfer mode (**a**), and electrical analogy of the thermal resistances (**b**), with φ being the total resistance against heat transfer, *T_1_* the ambient air temperature outside the tank, *T_2_* the wine temperature, *T_w1_* the temperature of the external wall of the tank and *T_w2_* the temperature of the inner wall, *h_c1_* the external convective coefficient, *h_c2_* the internal convective coefficient, and $\lambda_{w}$ the thermal conductivity of the tank wall whose thickness is denoted e.

In the case of large average diameter tanks, whose wall thickness is small compared to their diameter, the hypothesis of walls assimilated to a flat wall is realistic. The synoptic diagram of the heat transfer is shown in Figure 6a. When the outside and inside of the tank are at different temperatures, a heat supply (respectively a heat loss in the event of a negative thermal gradient) appears, slowed down by the presence of thermal resistances along its path. These thermal resistances take into account the thermo-physical properties of the internal and external fluids, the ventilation conditions of the storage areas of the tanks, but also the properties of the material constituting the tanks (thickness, thermal conductivity). The electrical analogy of the consideration of these thermal resistances is illustrated in Figure 6b. The total resistance against heat transfer φ will thus be considered as the arithmetic sum of the internal and external convective and conductive resistances.

The main heat source at the origin of the wine’s movement is the temperature of the inner surface of the tank, namely *T_w2_*, which is directly in contact with the wine. This parameter will be the thermal boundary condition of the numerical problem. Its determination is based on the conservation of the heat supply through the different environments crossed, expressed hereafter:

(4) $$\varphi =\frac{T_{1}-T_{2}}{\frac{1}{h_{c1}}+\frac{e}{\lambda_{w}}+\frac{1}{h_{c2}}}=h_{c2}\left(T_{w2}-T_{2}\right)$$

which leads to the following expression for T*_w2_*:

(5) $$T_{w2}=T_{2}+\frac{T_{1}-T_{2}}{1+h_{c2}\frac{\lambda_{w}+e\;h_{c1}}{\lambda_{w}\;h_{c1}}}$$

Knowing a priori the external and internal temperatures and the properties of the tank material, the determination of the temperature of the internal face of the tank *T_w2_* will be possible once the convective exchange coefficients have been accurately estimated. The respective convective coefficients are deduced from the Nusselt number *Nu*:

(6) $$\;\;h_{c1}=\frac{{\bar{Nu}}_{D}\;\lambda_{air}}{D};\;h_{c2}=\frac{{\bar{Nu}}_{H}\;\lambda_{wine}}{H}$$

with $\lambda_{air}$ and $\lambda_{wine}$ being the thermal conductivities of air and wine, respectively, *D* being the tank diameter, and *H* being the tank height.

For the internal convective coefficient *h_c2_* at the inside surface of the tank, we used the value determined in similar enological conditions [8], namely *h_c2_* = 30 W·m^−2^·K^−1^. For the external convective coefficient, which is a forced convection one, we assumed that the tank was exposed to a constant wind velocity of *V* ≈ 1 m·s^−1^ within the cellar. In such a case, the Nusselt number reported to the tank diameter in Equation (6) can be estimated as:

(7) $${\bar{Nu}}_{D}=0.32+0.43Re^{0.52}$$

with *Re* being the Reynolds number defined as:

(8) $$Re=\frac{VD}{\nu_{air}}$$

with $\nu_{air}$ being the kinematic viscosity of air (expressed in m^2^·s^−1^).

The Grashof number, being defined as the ratio of the buoyancy to viscous force acting on a fluid in the velocity boundary layer, is expressed hereafter for the wine stored in the tank:

(9) $$Gr_{H}=\frac{g\;\beta_{wine}\;\left(T_{w2}-T_{2}\right)H^{3}}{v_{wine}^{2}}$$

with g being the acceleration due to gravity, $\beta_{wine}$ the thermal expansion coefficient of wine, and $v_{wine}$ the kinematic viscosity of wine.

The thermo-physical characteristics of the materials used to determine the tank wall thermal boundary conditions are shown in Table 1.

For the internal free convection flow inside the tank, an analysis of the Rayleigh number (defined as $Ra_{H}=Gr_{H}\times Pr$) shows that the flow regime is laminar as $Ra_{H}<10^{9}$. For such a laminar regime, Kakaç et al. [9] reported the following empirical relationship for the Nusselt number:

(10) $${\bar{Nu}}_{H}=0.68+\frac{0.670\;Ra_{H}^{0.25}}{{\left[1+{\left(\frac{0.492}{Pr}\right)}^{9/16}\right]}^{4/9}}$$

Hence, we used the Eulerian multiphase model to simulate the two phases (wine and lees). In order to quantify the proportion of light lees remaining in suspension after alcoholic fermentation, small scale alcoholic fermentation was carried out in a laboratory on a sample of uncleared wine. The quantity of light lees was estimated to be about 0.03% of the total volume. The numerical procedure consisted of injecting the appropriate quantity of lees into the bottom of the tank and then allowing it to settle by sedimentation. The unsteady calculation started when the fluid was perfectly inert and the lees were completely settled at the bottom of the tank. Moreover, both wine and lees were initialized at a temperature of *T_wine_* = *T_lees_* = 12 °C. Gradually, a heat transfer by convection took place between the walls of the tank and the wine, resulting in a change in its density. In order to model as accurately as possible the slightest change in density as a function of temperature, we created a database specific to the thermo-physical properties of both wine and lees.

### 3.2. Equations and Numerical Scheme

The classical hydrodynamics in the liquid phase are described by the continuity and momentum conservation equations for laminar flows presented hereafter. The general form of the continuity equation can be written as:

(11) $$\frac{\partial \rho }{\partial t}+\nabla \times \left(\rho \vec{v}\right)=S_{m}$$

with *ρ* being the liquid density, $\vec{v}$ the velocity field and source $S_{m}$ being the mass added to the continuous phase from the dispersed second phase.

For a two-dimensional (2D) axisymmetric geometry, the continuity equation is given by:

(12) $$\frac{\partial \rho }{\partial t}+\frac{\partial }{\partial x}\left(\rho \nu_{x}\right)+\frac{\partial }{\partial r}\left(\rho \nu_{r}\right)+\frac{\rho \nu_{r}}{r}=S_{m}$$

with x being the axial coordinate, r the radial coordinate, $\nu_{x}$ the axial velocity and $\nu_{r}$ the radial velocity, respectively.

Conservation of momentum is described by:

(13) $$\frac{\partial }{\partial t}\left(\rho \vec{v}\right)+\nabla \times \left(\rho \vec{v}\vec{v}\right)=-\nabla p+\rho \vec{g}+\vec{F}$$

with p being the static pressure and $\rho \vec{g}$ and $\vec{F}$ being, respectively, the gravitational body force and external body forces (i.e., forces that arise from interaction between the liquid phase and the dispersed one).

For 2D axisymmetric geometries, the axial and radial momentum conservation equations are given by:

(14) $$\frac{\partial }{\partial t}\left(\rho \nu_{x}\right)+\frac{1}{r}\frac{\partial }{\partial x}\left(r\rho \nu_{x}\nu_{x}\right)+\frac{1}{r}\frac{\partial }{\partial r}\left(r\rho \nu_{r}\nu_{x}\right)\;=-\frac{\partial p}{\partial x}+\frac{1}{r}\frac{\partial }{\partial x}\left[r\mu \left(2\frac{\partial \nu_{x}}{\partial x}-\frac{2}{3}\left(\nabla \times \vec{v}\right)\right)\right]+\frac{1}{r}\frac{\partial }{\partial r}\left[r\mu \left(\frac{\partial \nu_{x}}{\partial r}+\frac{\partial \nu_{r}}{\partial x}\right)\right]+F_{x}$$

and

(15) $$\frac{\partial }{\partial t}\left(\rho \nu_{r}\right)+\frac{1}{r}\frac{\partial }{\partial x}\left(r\rho \nu_{x}\nu_{r}\right)+\frac{1}{r}\frac{\partial }{\partial r}\left(r\rho \nu_{r}\nu_{r}\right)\;=-\frac{\partial p}{\partial r}++\frac{1}{r}\frac{\partial }{\partial r}\left[r\mu \left(2\frac{\partial \nu_{r}}{\partial r}-\frac{2}{3}\left(\nabla \times \vec{v}\right)\right)\right]+\frac{1}{r}\frac{\partial }{\partial x}\left[r\mu \left(\frac{\partial v_{r}}{\partial x}+\frac{\partial \nu_{x}}{\partial r}\right)\right]-2\mu \frac{v_{r}}{r^{2}}+\frac{2}{3}\frac{\mu }{r}\left(\nabla \times \vec{v}\right)+F_{r}\;\;\;\;\;\;\;$$

where

(16) $$\nabla \times \vec{v}=\frac{\partial \nu_{x}}{\partial x}+\frac{\partial \nu_{r}}{\partial r}+\frac{\nu_{r}}{r}$$

with μ being the liquid phase dynamic viscosity.

### 3.3. Numerical Results

**Figure 7:**
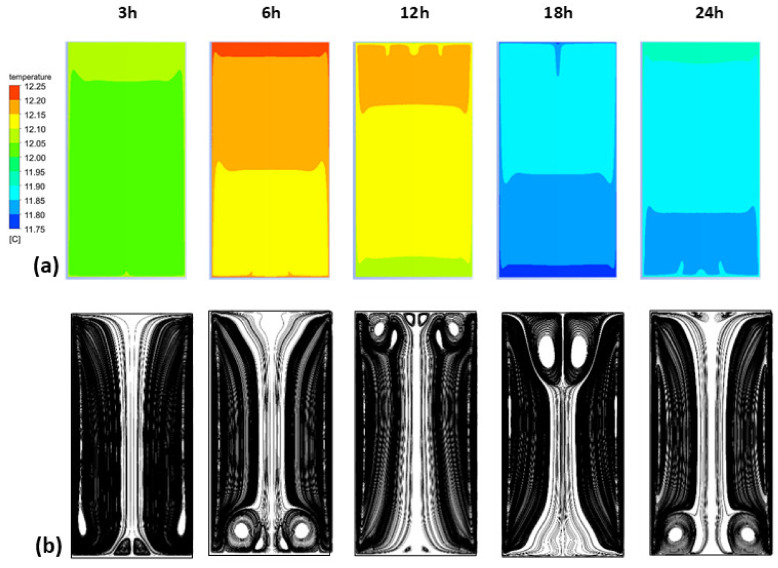
Evolution of the temperature of the wine over a 24 h cycle (**a**), and corresponding streamline patterns (**b**).

At the small scale of a 1 L vessel, laser tomography proved that low positive or negative thermal gradients between the wine (holding light lees) and its external environment could promote natural free convection and the subsequent resuspension of lees. To extrapolate these findings to full-scale tanks, a numerical study was carried out to simulate the two-phase flow behavior as a function of temperature variations over a 24 h cycle. Figure 7 shows the evolution of the temperature of the wine in the tank, and the corresponding streamline patterns at different times of the day (at 3, 6, 12, 18 and 24 h, respectively). Figure 7 a shows that the temperature of the wine evolves periodically according to the 24 h cycle displayed in Figure 5. However, it should be noted that the temperature variations remain low due to an important thermal inertia due to the volume of the tank. When the tank wall is heating up, the liquid near the wall also warms up by conduction, while the liquid in the center of the tank is still at the initial temperature (12 °C). As the density and viscosity of the wine vary with temperature, a slight thermal gradient between the wall and the wine is sufficient to set the wine in motion by a natural convection process. In the case of a heated wall, most of the heat flow is used to accelerate the liquid in the adjacent viscous layer [22]. Heating by conduction, at first dominant, is progressively supplanted by a convective heat transfer. Since the temperature of the tank wall varies continuously throughout the day, the topology of the wine flow will change accordingly.

Figure 7b shows the streamlines of the flow at different time intervals (3, 6, 12, 18 and 24 h), according to the 24 h cycle displayed in Figure 5. It can be noted that the topology of the flow patterns constantly varied throughout the day. The fluid motion resulting from the thermal gradient ensured a continuous and slow mixing of the entire tank volume. When the thermal gradient was positive (between 3–6 h and 18–24 h), the ascending flow operating along the side walls was then constrained to reorient at the top of the tank, and forced to descend along the vertical axis of symmetry. When the gradient was negative (between 6 and 18 h), the flow followed the wall from the top to the bottom of the tank and then rose from the center. Although similarities exist between the laboratory experiments and the numerical results, the tank geometry and timing were different, making comparison difficult. However, it must be noted that whatever the method, only a small thermal gradient is sufficient to set the entire liquid phase in motion.

**Figure 8:**
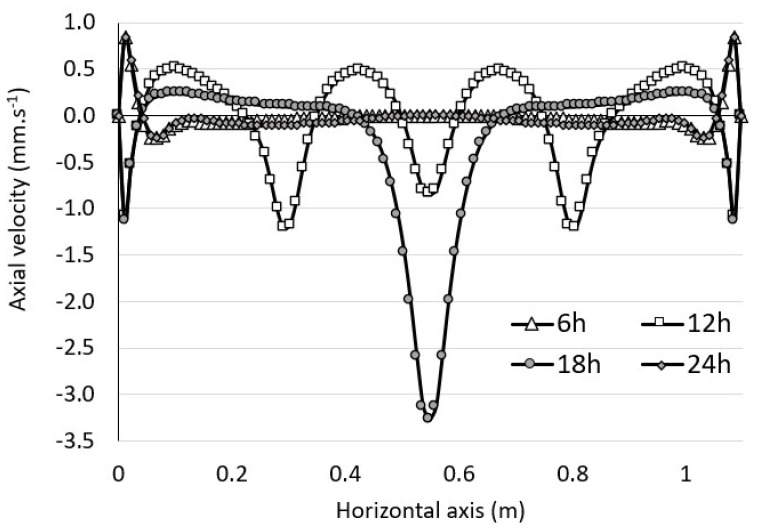
Axial velocity of the wine at 6, 12, 18 and 24 h, respectively, as plotted along a horizontal axis at y = 210 cm.

As shown in Figure 7b, the global motion consisted of an annular flow composed of several vortex rings whose locations and quantities varied continuously over time. Remarkable topological differences were noticeable, and several distinct situations were observed with the presence of a double, triple, quadruple or even quintuple vortex ring. Between 3 and 6 h, the thermal gradient was positive, and secondary vortex rings at the bottom of the tank were noted. Between 12 and 18 h, the thermal gradient was reversed and secondary vortex rings developed in the upper part of the tank, while they disappeared in the lower part. Then, the situation was reversed again from 18 h onwards. The flow dynamics were therefore strongly influenced by the unsteady temperature evolution of the wall. Figure 8 shows the evolution of the axial velocity of the wine plotted along a horizontal axis at y = 210 cm and for different times of the day (6, 12, 18 and 24 h). It is worth noting that the velocity of the wine was subjected to significant fluctuations over a 24 h cycle. Figure 8 shows that the flow close to the wall reversed its direction according to the positive or negative gradient (18, 24 h). In addition, the highest flow velocities (close to 3 mm·s^−1^) were observed at t = 18 h along the axis of symmetry of the tank. We can also note that the velocity profile for t = 6h merged with that of t = 24 h, transition time between positive and negative gradients.

### 3.4. Contribution of the Wine Mixing Dynamics on the Resuspension of Light Lees

The question that motivated this study was whether a low thermal gradient between the wine and the outer part of the tank would be sufficient to resuspend the light lees. We have shown that a low thermal gradient allows a continuous and slow mixing of the wine. The postprocessing of the CFD calculations allowed each phase of the flow (wine and lees) to be followed independently.

**Figure 9:**
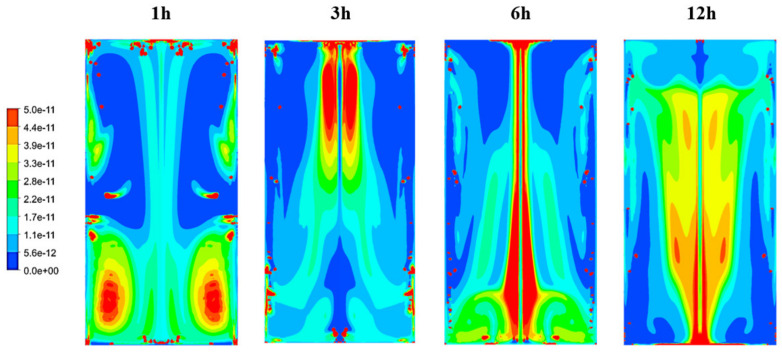
Time evolution of the volume fraction of lees throughout the first 12 h of the 24 h cycle (at 1 h, 3 h, 6 h and 12 h, respectively).

The time evolution of the volume fraction of lees in the tank, over a 12 h period, is displayed in Figure 9. We deliberately limited the volume fraction scale to highlight the concentration of lees in the fluid domain. As shown in Figure 9, the CFD calculations confirmed that the swirling movement of the liquid phase had sufficient energy to entrain the light lees and resuspend them in the wine bulk. The lees followed a similar path to the wine. They were first carried along by the natural convection flow that developed along the tank wall, hitting the top of the tank and then going down into the wine bulk.

**Figure 10:**
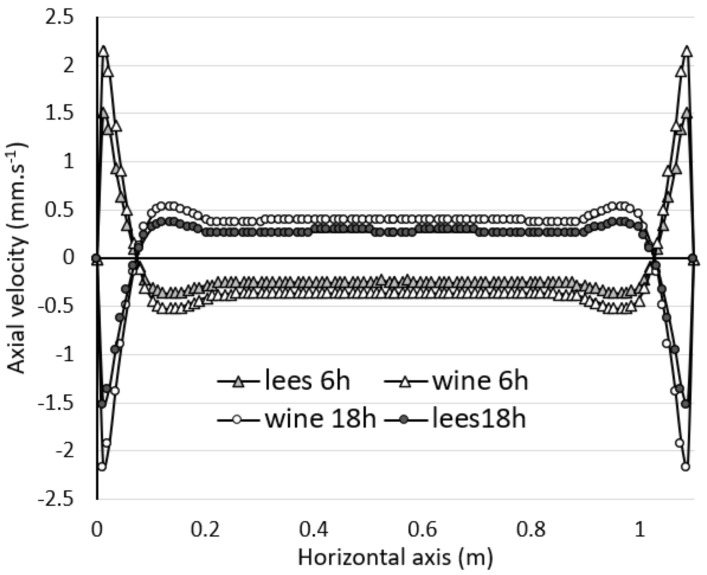
Comparison of the axial velocities of wine and lees, at t = 6 h and t = 18 h, as plotted along a horizontal axis, at y = 110 cm.

An interesting matter is the velocity of the lees in relation to that of the wine. Figure 10 shows the evolution of the axial velocities of the wine and the lees plotted along a horizontal axis for two different times (6 h and 18 h). The velocity curves for 6 h and 18 h exhibited a mirror symmetry due to a thermal gradient inversion. We can also see that at t = 6 h, the lees flow took the same path as the natural sedimentation direction in the central part of the tank, whereas they had an upward movement in the near wall. The finding was reversed at t = 18 h, with the positive velocity components becoming negative and vice versa. Although the sign of the axial velocity component was reversed, the velocities for t = 6 h and t = 18 h were of the same order of magnitude, because the temperature gradient was identical (+/−3 °C). In the near-wall zone, strongly influenced by the natural convection flow, the maximum lees velocity was about 1.5 mm·s^−1^, against 2.15 mm·s^−1^ for the wine. A key finding is that the average velocity of the wine was about 31% higher than that of the lees, which have a higher density. It should be noted, however, that the actual lees velocity was up to 250 times higher than the settling velocity previously determined using the Stokes equation (1). Moreover, it should be noted that the lees granulometry did not induce a sufficient sedimentation rate to oppose the vortex structures and the natural convection flow velocities involved. This implies that as long as the wine tank is not subjected to thermal equilibrium (identical temperature between the wine and the outside of the tank), the lees will not settle to the bottom of the tank. Finally, the numerical results confirmed that the laboratory results can be extrapolated to full-scale tanks, and that a small thermal gradient is sufficient to resuspend the light lees.

## 4. Conclusions

This study shows for the first time how the resuspension of light lees may occur in wine tanks when the two-phase flow mixing has a thermal origin rather than a mechanical one, such as *bâtonnage*. This resuspension, if not desired, can have harmful effects on the clarification or filtration processes of wines. To ensure that our observations made at the laboratory scale (i.e., in a 1 L vessel) could be extended to the scale of real tanks, numerical simulations were performed using a CFD method. This study yielded three major findings. Firstly, it was shown that low thermomechanical energy is sufficient to set the liquid phase in vortex motion, regardless of the sign of the thermal gradient. Secondly, it was shown that the granulometry of the lees did not induce a sufficient sedimentation rate to oppose the vortex structures and the natural convection flow velocities involved. The higher the thermal gradient between the outside and inside of the container, the higher the quantity of lees that was resuspended. And thirdly, our numerical results evidenced that the average velocity of the wine is about 31% higher than that of the lees, which have a higher density. Simulations carried out on a real size cylindrical geometry tank for a positive and negative gradient confirmed the observations made during laboratory experiments. Finally, both methods evidenced that even under low thermal gradients, the resuspension of light lees occurs and follows the flow features.

This study was conducted in the Champagne region, where Chardonnay, Pinot noir and Pinot Meunier grape varieties are grown; however, it could easily be extended to the large variety of wines elaborated throughout the world, including those with different alcohol and sugar levels. The various proportions of ethanol and sugars will indeed modify both the density and viscosity of the wine matrix, and therefore, the subsequent flow patterns dynamics in the tank resulting from thermal gradients will vary. This may be examined in future work.

## Figures and Tables

**Table 1:** Thermo-physical properties of the various materials, with *Pr* defined as the ratio of kinematic viscosity to thermal diffusivity.

	Kinematic Viscosity, ν (m^2^·s^−1^)	Thermal Conductivity, λ (W·m^−1^·K^−1^)	Thermal Expansion Coefficient, β (K^−1^)	Prandtl Number, Pr
Stainless steel	—	16	—	—
Air	1.57·10^−5^	0.0262	—	0.7
Wine	1.25·10^−6^	0.46	8·10^−4^	9.4

## Nomenclature

D	Diameter of the tank, m
$d_{l}$	Average lees diameter, m
e	Wall tank thickness, m
$\vec{F}$	External body forces, N
Gr	Grashof number
*H*	Height of the tank, m
$h_{c1}$	Convective heat transfer coefficient of air, W·m^−2^·K^−1^
$h_{c2}$	Convective heat transfer coefficient of wine, W·m^−2^·K^−1^
*Nu*	Nusselt number
p	Static pressure, Pa
Pr	Prandtl number
r	Radial coordinate
Ra	Raleigh number
$S_{m}$	Mass added to the continuous phase from the dispersed second phase
*T_w1_*	Temperature of the outer surface of the tank, K
*T_w2_*	Temperature of the inner surface of the tank, K
*T_1_*	Air temperature, K
*T_2_*	Wine temperature, K
$\nu_{x}$	Axial velocity, m·s^−1^
$\nu_{r}$	Radial velocity, m·s^−1^
x	Axial coordinate
$\lambda_{air}$	Thermal conductivity of air, W·m^−1^·K^−1^
$\lambda_{wine}$	Thermal conductivity of wine, W·m^−1^·K^−1^
$\mu_{w}$	Dynamic viscosity of wine, kg·m^−1^·s^−1^
$\rho_{l}$	Density of lees, kg·m^−3^
$\rho_{w}$	Density of wine, kg·m^−3^
*ϕ*	Heat flux, W·m^−2^
ρ	Density, kg·m^−3^
g	Gravitational acceleration, m·s^−2^
*ν*	Kinematic viscosity, m^2^·s^−1^
*β*	Thermal expansion coefficient, K^−1^
